# THE “DOUBLE JEOPARDY” OF MIDLIFE AND OLD AGE MORTALITY TRENDS IN THE UNITED STATES

**DOI:** 10.1093/geroni/igad104.3625

**Published:** 2023-12-21

**Authors:** Leah Abrams, Neil Mehta, Mikko Myrskyla

**Affiliations:** Tufts University, Medford, Massachusetts, United States; University of Texas Medical Branch, Galveston, Texas, United States; Max Planck Institute, Rostock, Mecklenburg-Vorpommern, Germany

## Abstract

Since 2010, U.S. life expectancy growth has stagnated. Much research on U.S. mortality has focused on working age adults given adverse trends in drug overdose deaths, other external causes of death, and cardiometabolic deaths in midlife. We show that the adverse mortality trend at retirement ages (65+ years) has in fact been more consequential to the U.S. life expectancy stagnation since 2010, as well as excess deaths and years of life lost in 2019, than adverse mortality trends at working ages. These results reveal that the U.S. is experiencing a “double jeopardy” that is driven by both mid-life and older-age mortality trends, but more so by older-age mortality. Understanding and addressing the causes behind the worsening mortality trend in older ages will be essential to returning to the pace of life expectancy improvements that the U.S. had experienced for decades.

